# A Novel Prognostic Model Based on Seven Necroptosis-Related miRNAs for Predicting the Overall Survival of Patients with Lung Adenocarcinoma

**DOI:** 10.1155/2022/3198590

**Published:** 2022-03-24

**Authors:** Xiaohua Hong, Guangyao Wang, Kai Pei, Chunmei Mo, Zhen Rong, Guanglan Xu

**Affiliations:** ^1^Graduate School, Guangxi University of Chinese Medicine, Nanning 530000, China; ^2^Department of Respiratory and Critical Care Medicine, The First Affiliated Hospital of Guangxi University of Chinese Medicine, Nanning 530000, China; ^3^Department of Oncology, Bao'an Authentic TCM Therapy Hospital, Shenzhen 518038, China

## Abstract

Lung adenocarcinoma (LUAD) remains one of the leading causes of cancer-related deaths worldwide. This study is aimed at constructing a risk scoring model based on necroptosis-related miRNAs to predict prognosis of LUAD. Expression profile of miRNA in LUAD was downloaded from The Cancer Genome Atlas (TCGA) database. We screened the differentially expressed necroptosis-related miRNAs between LUAD patients and normal samples, thus constructed a seven miRNA-based risk stratification on the basis of the TGCA cohort. This risk stratification was prove to be effective in predicting the overall survival (OS) of patients with LUAD. Furthermore, we constructed a nomogram model based on the combination of risk characteristics and clinicopathological features, which was also prove to be accurate and efficient in predicting OS of LUAD patients. Functional enrichment analyses on the targeted genes of these miRNAs with prognostic value were carried out. Results indicated that these targeted genes were closely related to the development and metastasis of tumors. In summary, our research has developed a prognostic model based on the expression of miRNAs related to necroptosis. This model might be used to predict the prognosis of LUAD accurately, which might be helpful in improving treatment efficacy of LUAD.

## 1. Introduction

Lung cancer is a common but complex malignant disease, and most patients with lung cancer were diagnosed at advanced stage [1]. According to the latest global cancer statistics report by the International Agency for Research on Cancer, lung cancer remains the leading cause of cancer death worldwide, 2.2 million people were diagnosed with lung cancer in 2020 worldwide, and 1.79 million were died of lung cancer [2]. Unfortunately, the morbidity and mortality of lung cancer are still rising globally [3]. Lung adenocarcinoma (LUAD) represents the most frequent pathological type in lung cancer, which accounts for about 40% of the total lung cancers [4, 5]. Although advances have been made in current treatment strategies for lung cancer (surgical resection, chemotherapy, radiotherapy, molecular targeted therapy, and immunotherapy), the prognosis of lung cancer is still not satisfied. The incidence of 5-year survival of patients with lung cancer remains only 4%-17%, and the incidence of 5-year survival is less than 5% in patients with metastasized tumor [6–8]. Therefore, early detection with a comprehensive and accurate risk assessment is of great significance in diagnosing and monitoring of lung cancer. Current tools of risk assessment and monitoring of lung cancer are mostly based on clinical characteristics and pathological parameters, in which TNM stratification is most commonly used. However, the existing TNM model is often associated with a limited predictive confidence in predicting prognosis of lung cancer, which was comprised of huge heterogeneity among individuals. Therefore, there is a unmet need for cooperating clinicopathological characteristics of the genome in assess individual survival prognosis.

Necroptosis is a newly identified caspase-independent function of programmed cell death, which is different from apoptosis [9]. Morphological manifestion of necroptosis is demonstrated by swelling and rounding cells, explosive plasma membrane rupture, mitochondrial dysfunction and loss of mitochondrial membrane potential, and cell membrane perforation [10]. When the expression of caspase-8 is inhibited or at a low level in cells, receptor-interacting protein 1 (RIP1) can recruit receptor-interacting protein 3 (RIP3) to form a RIP1-RIP3 complex, which induces a mixed lineage of pseudokinases. The kinase domain-like protein (MLKL) is phosphorylated to form necrosomes, which leads to necroptosis [11]. Necroptosis has been proved to have a double-edged sword effects on cancer, and the relationship between necroptosis and cancer is complicated. Although it is reported that necroptosis can inhibit tumor development and metastasis when apoptosis is blocked, its key regulators will also provide assistance for tumor metastasis and progression [12]. Emerging evidences have indicated that necroptosis can inhibit tumor development and metastasis, thus may be utilized as a potential method in treating cancers [13–15]. For example, necroptotic MiR-7-5p inhibits tumor metastasis by targeting NOVA2 in lung cancer [16]. Although previous studies have reported that necroptosis has antitumor effects in various types of cancer including LUAD, the knowledge of the prognostic value of necroptosis-related microribonucleic acid (miRNA, a set of short noncoding RNA with regulatory functions [17]) is still poor.

In this study, we constructed a risk scoring stratification based on miRNAs related to necroptosis. Meanwhile, to improve the prognosis confidence of this model, we have established a nomogram that integrates risk scores and clinical factors. This study is aimed at evaluating the prognostic value of necroptosis-related miRNAs in LUAD and develops a prognostic nomogram based on necroptosis-related miRNAs as a accurate prediction tool to effectively evaluate the clinical prognosis of patients with LUAD.

## 2. Materials and Methods

### 2.1. Data Collection

**Figure 1:**
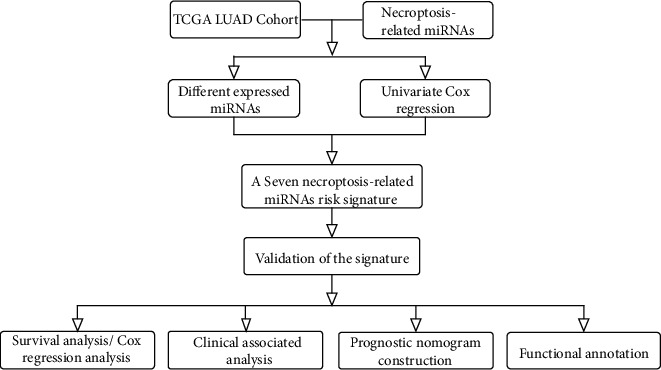
Flow diagram of the study.

The schematic diagram was shown in Figure 1. Transcriptomic data sets of miR-seq were downloaded from the public lung adenocarcinoma (LUAD) database of TCGA (https://portal.gdc.cancer.gov/), which contain 521 LUAD patient samples and 46 normal control samples. Clinical data of corresponding patients was also downloaded. We excluded data from patients with no OS information. Thus, a total of 499 LUAD patients were included in the subsequent analyses. Detailed information on these 499 patients with LUAD is presented in Supplementary Table 1. This research complies with the TCGA data access policy. Meanwhile, this study was exempted from relevant ethical approval because the TCGA database is publicly available.

### 2.2. Differentially Expressed Necroptosis-Related miRNAs between LUAD Patients and Normal Control Samples

Thirteen miRNAs related to necroptosis were profiled according to the previous publication [12] (miR-495, miR-331-3p, miR-15a, miR-148a-3p, miR-7-5p, miR-141-3p, miR-425-5p, miR-200a-5p, miR-210, miR-223-3p, miR-500a-3p, miR-181-5p, miR-16-5p). By using the “tidyverse” *R* package, the expression of 13 necroptosis-related miRNAs was profiled with data downloaded from the TCGA database and was used for subsequent analysis. Then, the “limma” *R* package was used to identify the differentially expressed necroptosis-related miRNAs between LUAD patient samples and normal control samples, with false discovery rate (FDR) < 0.05 as statistically significant.

### 2.3. Establishment of a Prognostic Stratification Based on the TCGA Cohort

In order to assess the prognostic value of these necroptosis-related miRNAs, we used a Cox regression analysis to identify the miRNAs significantly related to the OS of LUAD. A total of 7 necroptosis-related miRNAs were identified to be significantly related to OS. Due to the limited number of identified miRNAs (less than 10), no further LASSO Cox regression analysis was performed. At last, 7 necroptosis-related miRNAs were retained to establish a risk scoring model. The risk score of each patient is calculated as the sum of the remaining scores of each miRNA, and each miRNA score is calculated by multiplying the miRNA coefficient by the miRNA expression level. The specific risk score formula is as follows: risk score = ∑_*i*=1_^*n*^coefi × expi (*n* represents the number of miRNAs, coefi represents the regression coefficient of miRNAi, and expi represents the expression level of miRNAi) [18, 19]. According to the median value of the risk score, TCGA LUAD patients were divided into high- and low- risk groups. The “survival” *R* package, “timeROC” *R* package, and “ROCR” *R* package were used to compare the OS of the high- and low-risk groups, and 3-year and 5-year ROC curve analysis was performed. In addition, to investigate whether the risk score could be used as an independent factor of OS in LUAD, univariate and multivariate Cox regression analysis was performed. Meanwhile, the “pheatmap” package was utilized to plot a heat map of risk characteristics and related clinical factors.

### 2.4. Establishment of the Forecastive Nomogram

The “survival” *R* package and the “rms” *R* package were used to construct a predictive nomogram model, which includes risk scores and associated clinical factors (age, gender, smoking history (years), and TNM staging). A calibration curve was established to evaluate the consistency between the results of predictive model and the actual clinical results when nomogram model was applied.

### 2.5. Functional Enrichment Analysis

Potential targeted genes of these miRNAs were identified by taking the intersection of predicted genes by miRDB (http://www.mirdb.org/), miRTarBase (http://mirtarbase.mbc.nctu.edu.tw/php/index.php), and TargetScan (http://www.targetscan.org). Identified targeted genes were anaylyzed using gene ontology (GO) and Kyoto Encyclopedia of Genes and Genomes (KEGG). The whole list of the targeted genes is in Supplementary Table 2.

## 3. Results

### 3.1. Differentially Expressed Necroptosis-Related miRNAs between Tumor and Normal Samples

**Figure 2:**
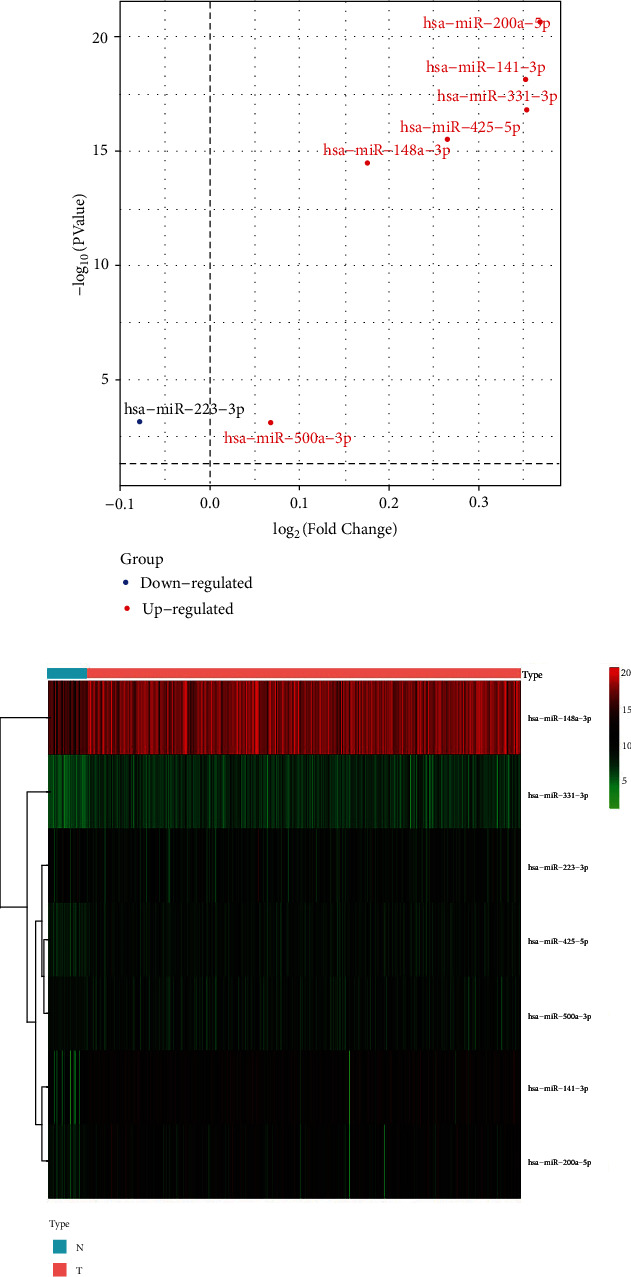
Differentially expressed necroptosis-related miRNAs. (a) A volcano plot of expression change comparing the tumor and normal samples, with the seven downregulated and upregulated miRNAs. (b) The notably differentially expressed necroptosis-related miRNA in each biological specimen from TCGA LUAD cohort were displayed by heat map.

A total of 7 differentially expressed miRNAs were identified between tumor and normal samples, among which miR-141-3p, miR-148a-3p, miR-200a-5p, miR-331-3p, miR-425-5p, and miR-500a-3p were upregulated in the cancer group, while miR-223-3p was downregulated (Figure 2(a)). The expression level of differentially expressed miRNAs was shown by a heat map. Rows represented miRNAs, and columns represented biological samples. The red color represented higher expression, and the green color represented lower expression (Figure 2(b)).

### 3.2. Establishment of a Prognostic Model Based on the TCGA Cohort

**Figure 3:**
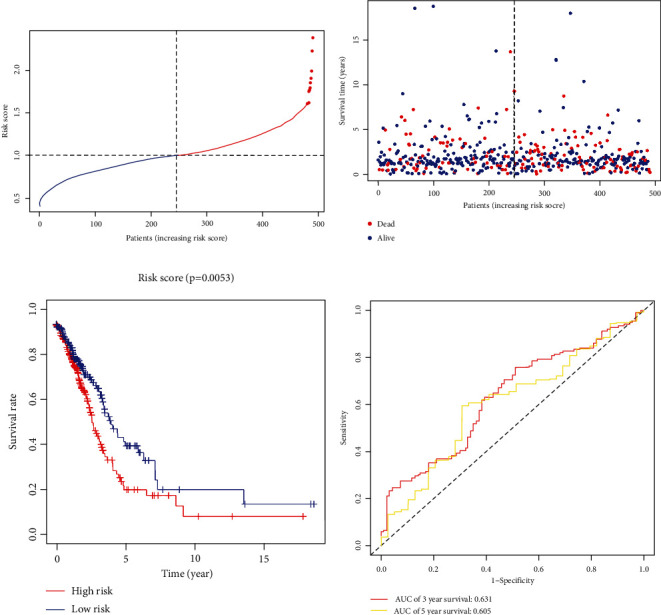
Construction of the prognostic signature based on the TCGA discovery cohort. (a) The distribution of risk scores. (b) The distribution of OS and OS status in the high- and low-risk score groups. (c) The distribution of OS and OS status in the high- and low-risk groups. (d) Kaplan–Meier curves for the OS of patients in the high- and low-risk groups.

Cox regression analysis was used to identify those necroptosis-related miRNAs which were significantly related to OS of LUAD. A total of 7 necroptosis-related miRNAs were identified and were used to establish a risk scoring model. Formula of scoring for each individual was (−0.03043 × miR − 141 − 3p)(−0.14387 × miR − 148a − 3p)(−0.02951 × miR − 200a − 5p)(0.07482 × miR − 223 − 3p)(0.01450 × miR − 331 − 3p)(0.18310 × miR − 425 − 5p)(−0.11271 × miR − 500a − 3p). LUAD patients were assigned to the high- or low-risk groups, with each group contains 245 patients (Figure 3(a)).

As presented in the figure of the survival status of each patient (left side of the dotted line represented the low-risk group, and right side of the dotted line represented the high-risk group), patients in the low-risk group were associated with lower incidence of deaths and longer OS compared to patients in the high-risk group (Figure 3(b), left side of the dotted line). The survival curve showed that OS of patients in the low-risk group was longer than patients in the high-risk group in the TCGA LUAD cohort (*P* = 0.0053, Figure 3(c)). Sensitivity and specificity of the prognostic model were calculated using a ROC method. Results revealed that the AUC of 3-year and 5-year OS was 0.631 and 0.605, respectively (Figure 3(d)).

### 3.3. Independent Prognostic Value of Risk Factors

**Figure 4:**
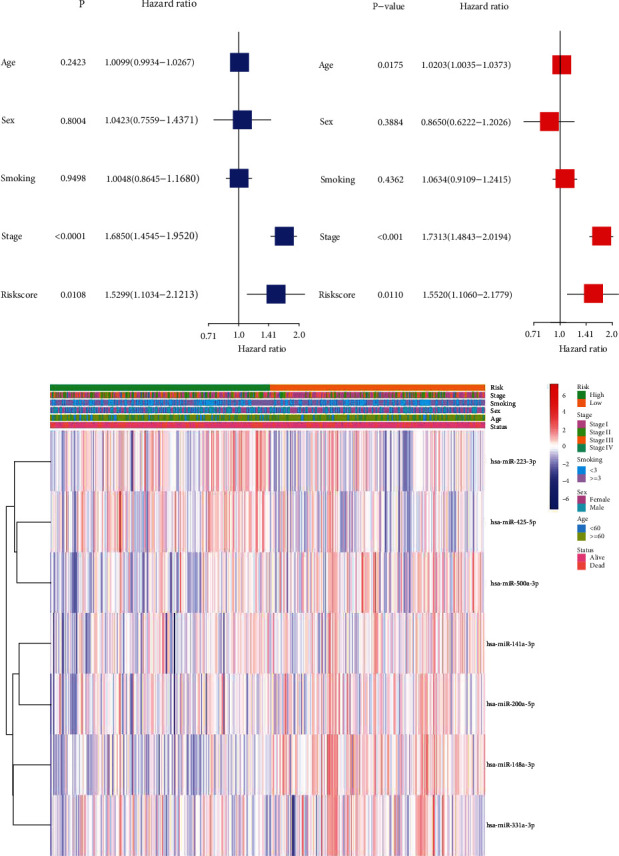
Univariate and multivariate Cox regression analyses for the risk score. (a) Univariate analysis for the TCGA cohort. (b) Multivariate analysis for the TCGA cohort. (c) Heatmap of clinicopathological characteristics of the low- and high-risk patients.

To evaluate whether these miRNA-based risk scores could be used as independent prognostic factors in the TCGA cohort, univariate and multivariate Cox regression analysis was performed. Results of univariate Cox regression analysis showed that the 7 necroptosis-related miRNA risk scores were independent prognosis factors in the TCGA cohort (HR = 1.5299, 95% CI [1.1034-2.1213], *P* value =0.0108 (*P* < 0.05), Figure 4(a)). Meanwhile, results of multivariate Cox regression analysis also confirmed that the risk scores were prognostic factors for LUAD patients after integrating other confounding factors (HR = 1.5520, 95% CI [1.1060-2.1779], *P* value =0.0110 (*P* < 0.05), Figure 4(b)).

### 3.4. Establishment of Risk Features and Clinicopathological Characteristic Combined Nomogram

**Figure 5:**
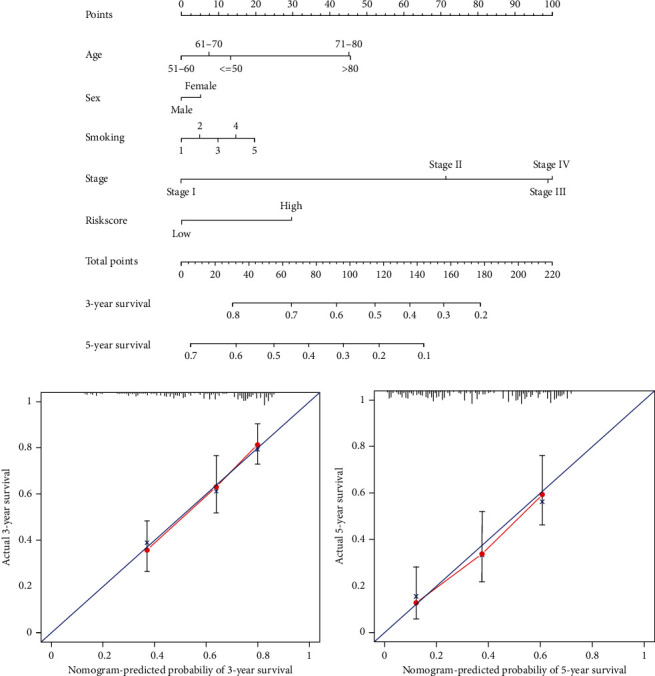
Integrated prognostic nomogram by combining risk signature and clinicopathological features. (a) A nomogram predicting 3- and 5-year OS of LUAD. (b) The calibration plots demonstrated that the nomogram showed excellent performance for predicting the 3-year OS. (c) The calibration plots demonstrated that the nomogram showed excellent performance for predicting the 5-year OS.

Multiple factors and the risk value of each patient were integrated and calculated using a visualized nomogram. This nomogram combined risk characteristics and clinicopathological characteristics, which included five factors as risk characteristics, age, gender, smoking history (years), and disease stage and was used to predict the 3-year and 5-year OS of LUAD patients. According to the score value corresponding to each factor on the scale, a total score from the nomogram according to the individual situation was calculated for each patient, and the total score was used to predict the 3-year and 5-year OS (Figure 5(a)). Confidence of the nomogram was confirmed by the calibration chart, which indicated that the nomogram was associated with a ideal performance in predicting the 3-year and 5-year OS of LUAD patients (Figures 5(b) and 5(c)).

### 3.5. GO Enrichment and KEGG Pathway Analysis

**Figure 6:**
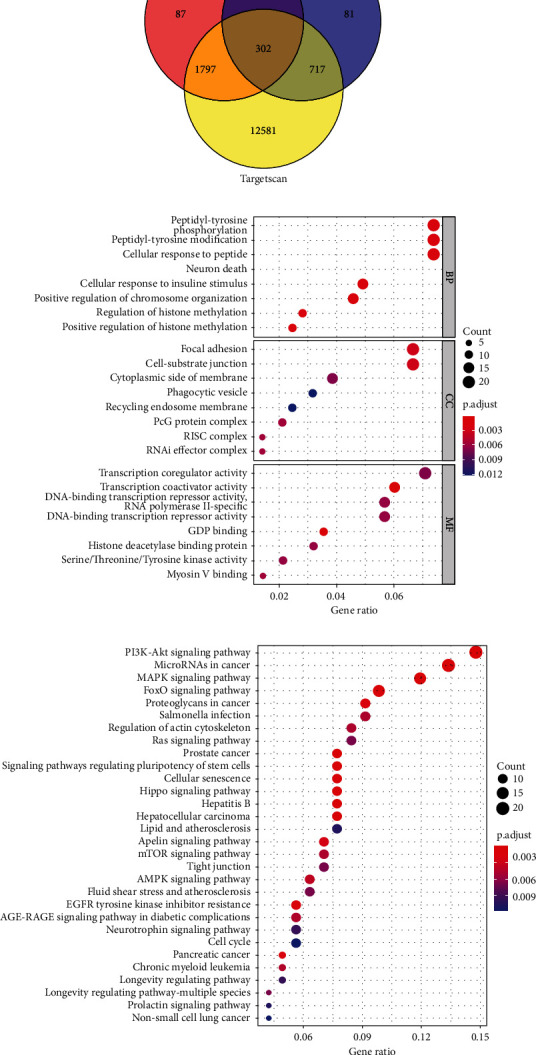
Functional annotation of the risk signature. (a) Venn diagram for the shared genes among three online databases. (b) The results of GO enrichment analysis. (c) The results of KEGG pathway analysis.

A total of 302 targeted genes of these miRNAs with potential prognostic value were identified by Venn analysis using miRDB, miRTarBase, and TargetScan databases (Figure 6(a)). The whole list of the targeted genes is in Supplementary Table 2. GO enrichment and KEGG pathway analysis of these targeted genes were further performed. The top 8 enriched biological processes, cell components, and molecular functions with the highest reliability based on the *P* value were shown in Figure 6(b).

PI3K − Akt signaling pathway, microRNAs in cancer, MAPK signaling pathway, FoxO signaling pathway, and proteoglycans in cancer were the most significantly enriched pathways. Illustrated by the KEGG analysis, these targeted genes of miRNAs were mainly related to cell proliferation, migration, invasion, and autophagy (Figure 6(c)).

## 4. Discussion

Necroptosis represents a form of cell death which is associated with the morphological features of necrotic cells, as well as intrinsic signal transductions similar to that of apoptotic cells. However, necroptosis differs from apoptosis, and it contributes to inhibition of tumor proliferation and metastasis [20–22]. Since the term necroptosis was proposed, a large number of studies have focusing on it. However, the prognostic value of necroptosis-related miRNAs on LUAD has not been fully established. In this present study, we investigated the prognostic value of 13 necroptosis-related miRNAs and their potential regulatory mechanisms in LUAD. By profiling the expression levels of 13 known necroptosis-related miRNAs in LUAD patients and normal control tissues, we found that most miRNAs (7/13) were differentially expressed between LUAD patients and normal control tissues. To evaluate the prognostic value of these necroptosis-related miRNAs, a Cox regression analysis was used to construct a risk stratification of 7 necroptosis-related miRNAs. Results indicated that this miRNA-based stratification was associated with significant value in distinguishing patients with low or high risk. In addition, based on the 7 necroptosis-related miRNA risk stratification and clinicopathological characteristics, we established a nomogram in the LUAD cohort. This nomogram was shown to be associated with significant superiority in accurately predicting OS of LUAD comparing to conventional evaluation tools (such as TNM staging) and promoted the clinical application of the necroptosis-related miRNA risk stratification to a large extent.

By using the GO enrichment and KEGG pathway analysis, we further investigated the molecular functions underlying these necroptosis-related miRNAs. Our results indicated that targeted genes of these necroptosis-related miRNAs were mainly related to functions of cell proliferation, focal adhesion, cell matrix adhesion, and cell aggregation adhesion, which were reported to be closely associated to tumor cell migration and tumor metastasis. [23–25]. Therefore, targeted therapy by inhibiting these cell-cell interactions may represent an important therapeutic strategy to improve the outcome of LUAD treatment. According to our finding, the most enriched PI3K-Akt signaling pathway was considered to be the key signal of cell proliferation, migration, and even chemotherapy resistance in lung cancer cells [26, 27]. In addition, the MAPK signaling pathway and Ras signaling pathway were also reported to be closely related to the regulation of lung cancer cell proliferation, differentiation, and migration [28, 29]. Inhibition of these pathways may contribute to the inhibition of the growth and metastasis of lung cancer and may help improve the prognosis of LUAD.

Mechanistically, the risk hazard ratio of miR-223-3p was 1.07770 in this study, suggesting that the miR-223-3p overexpression was related to the poor prognosis of LUAD. Studies have shown that this miRNA plays a tumor-promoting effect in several types of cancers asides lung cancer. For example, upregulation of miR-223-3p in non-small-cell lung cancer enhances tumor cell viability, migration, and invasion. High expression of this miRNA is related to a significant low survival rate and inferior outcomes of patients [30]. Similar phenomenons were also reported by Liu et al. and other [31]. In addition, the level of upregulation of miR-223-3p was positively correlated with the depth of tumor invasion and the degree of lymph node metastasis in gastric cancer [32]. All these observations have indicated that miR-223-3p was associated with an inferior prognosis. The risk characteristic hazard ratio of miR-425-5p in this study was also greater than 1, which was 1.20093, suggesting that the high expression of miR-425-5p may be associated with a cancer-promoting effect. A recent study found that miR-425-5p is overexpressed in lung cancer tissues, which enhances lung cancer cell proliferation and colony formation. It was also found that miR-425-5p promotes the development of lung cancer through the PTEN/PI3K/AKT signal axis [33]. In addition, the elevated expression of miR-425-5p significantly promoted the invasion and metastasis of gastric cancer and was an independent prognostic factor for a poor recurrence-free survival in patients with GC [34].

The risk characteristic hazard ratio of miR-141-3p in our study was 0.97003, indicating that miR-141-3p was a protective miRNA for LUAD. Studies have confirmed that the expression level of miR-141-3p in non-small-cell lung cancer tissues is downregulated, and its low expression is associated with poor prognosis, suggesting that miR-141-3p may be a tumor suppressor, and the high expression of this factor can help inhibit non-small-cell lung cancer cells The development of [35].

In this study, the risk characteristic hazard ratios of miR-148a-3p and miR-500a-3p were also less than 1, which were 0.86600 and 0.89341, respectively, suggesting that miR-148a-3p and miR-500a-3p were also protective miRNAs. These observations are consistent with previous studies. Studies by Xie et al. and Bai et al. both confirmed that miR-148a-3p was significantly downregulated in non-small-cell lung cancer, and its overexpression can inhibit the proliferation and epithelial-mesenchymal transition of non-small-cell lung cancer and plays an inhibitory role on the tumor cells [36, 37]. Compared with normal tissues, the expression level of miR-500a-3p in lung cancer tissues was significantly reduced, and low expression level of miR-500a-3p was closely related to the adverse clinical outcome of lung cancer [38]. The risk characteristic hazard ratio of miR-200a-5p in our study was 0.97092, suggesting that miR-200a-5p may be a tumor suppressor factor.

There are no functional study identifying the role of miR-200a-5p in LUAD. A number of studies [39, 40] have shown that miR-331-3p plays an inhibitory role in tumor proliferation, migration, and invasion. However, the risk characteristic hazard ratios of miR-331-3p in our study was higher than 1, indicating that the miR-331-3p overexpression might be related to poor prognosis, which was in contrary to previous studies. However, owing to the fact that the modulatory effects of miRNAs are impacted by tumor microenvironment, the role of specific miRNA in different types of cancer may be inconsistent or even the on the opponent site [41]. It is necessary to continue to investigate the effect of miR-331-3p in LUAD. To overcome limitations of this present study, large-scale independent research is needed to verify the effectiveness of this risk stratification in the future.

## 5. Conclusion

We analyzed the prognostic value of necroptosis-related miRNAs in LUAD for the first time. Generally, 13 necroptosis-related miRNAs to were screened, and 7 miRNAs were identified to be associated with significant prognostic value. These miRNAs play an important role in the development and metastasis of LUAD and may be potential therapeutic targets for LUAD. In addition, the nomogram, which was established by the combination of risk characteristics and clinicopathological characteristics in the LUAD cohort, could effectively predict the clinical prognosis of LUAD and provide an accurate and individualized prediction tool for clinic use. In summary, this study helps to understand the biological behavior and potential treatment targets of LUAD from the perspective of necroptosis.

## Data Availability

Transcritpional data sets of miR-seq were downloaded from the public lung adenocarcinoma (LUAD) database of TCGA (https://portal.gdc.cancer.gov/), which contain 521 LUAD patient samples and 46 normal control samples. Clinical data of corresponding patients was also downloaded. Potential targeted genes of these miRNAs were identified by taking the intersection of predicted genes by miRDB (http://www.mirdb.org/), miRTarBase (http://mirtarbase.mbc.nctu.edu.tw/php/index.php), and TargetScan (http://www.targetscan.org).

## Supplementary Materials

Supplementary 1 Table S1: detailed information on these 499 patients with LUAD.

Supplementary 2 Table S2: the detailed results of the targeted genes.
