# Supplementary material for: A Novel Prognostic Model Based on Seven Necroptosis-Related miRNAs for Predicting the Overall Survival of Patients with Lung Adenocarcinoma
Source: Biomed Res Int. 2022 Mar 24;2022:3198590. doi: 10.1155/2022/3198590 (PMC8972154; doi:10.1155/2022/3198590)
Supplement: Supplementary 2 — Table S2: the detailed results of the targeted genes. [file 3198590.f2.docx]

**Table S2. The detailed results of the targeted genes**

| Gene | Description | HGNC ID |
| --- | --- | --- |
| TNRC6B | Trinucleotide Repeat Containing Adaptor 6B | 29190 |
| CLOCK | Clock Circadian Regulator | 2082 |
| ADARB1 | Adenosine Deaminase RNA Specific B1 | 226 |
| BICD2 | BICD Cargo Adaptor 2 | 17208 |
| BRD3 | Bromodomain Containing 3 | 1104 |
| TRAM1 | Translocation Associated Membrane Protein 1 | 20568 |
| BAP1 | BRCA1 Associated Protein 1 | 950 |
| ASXL1 | ASXL Transcriptional Regulator 1 | 18318 |
| BAG2 | BAG Cochaperone 2 | 938 |
| KLF11 | Kruppel Like Factor 11 | 11811 |
| TNKS2 | Tankyrase 2 | 15677 |
| KLF12 | Kruppel Like Factor 12 | 6346 |
| WRB(GET1) | Guided Entry Of Tail-Anchored Proteins Factor 1 | 12790 |
| WWTR1 | WW Domain Containing Transcription Regulator 1 | 24042 |
| VPS37A | VPS37A Subunit Of ESCRT-I | 24928 |
| C10orf88 | Chromosome 10 Open Reading Frame 88 | 25822 |
| ZEB1 | Zinc Finger E-Box Binding Homeobox 1 | 11642 |
| ZEB2 | Zinc Finger E-Box Binding Homeobox 2 | 14881 |
| NRP1 | Neuropilin 1 | 8004 |
| NRSN2 | Neurensin 2 | 16229 |
| OR2A4 | Olfactory Receptor Family 2 Subfamily A Member 4 | 14729 |
| TNFRSF10B | TNF Receptor Superfamily Member 10b | 11905 |
| PAPPA | Pappalysin 1 | 8602 |
| KLF6 | Kruppel Like Factor 6 | 2235 |
| LBR | Lamin B Receptor | 6518 |
| UBXN7 | UBX Domain Protein 7 | 29119 |
| OGT | O-Linked N-Acetylglucosamine (GlcNAc) Transferase | 8127 |
| CDC25B | Cell Division Cycle 25B | 1726 |
| CDK19 | Cyclin Dependent Kinase 19 | 19338 |
| CERS6 | Ceramide Synthase 6 | 23826 |
| CLEC16A | C-Type Lectin Domain Containing 16A | 29013 |
| WNK1 | WNK Lysine Deficient Protein Kinase 1 | 14540 |
| PTPRD | Protein Tyrosine Phosphatase Receptor Type D | 9668 |
| YAP1 | Yes1 Associated Transcriptional Regulator | 16262 |
| YWHAG | Tyrosine 3-Monooxygenase/Tryptophan 5-Monooxygenase Activation Protein Gamma | 12852 |
| ZBTB18 | Zinc Finger And BTB Domain Containing 18 | 13030 |
| ZC3H4 | Zinc Finger CCCH-Type Containing 4 | 17808 |
| DCUN1D3 | Defective In Cullin Neddylation 1 Domain Containing 3 | 28734 |
| DPY19L1 | Dpy-19 Like C-Mannosyltransferase 1 | 22205 |
| DSTYK | Dual Serine/Threonine And Tyrosine Protein Kinase | 29043 |
| E2F3 | E2F Transcription Factor 3 | 3115 |
| ELAVL2 | ELAV Like RNA Binding Protein 2 | 3313 |
| ELAVL4 | ELAV Like RNA Binding Protein 4 | 3315 |
| EPHA2 | EPH Receptor A2 | 3386 |
| EPHA7 | EPH Receptor A7 | 3390 |
| TXNIP | Thioredoxin Interacting Protein | 16952 |
| CCDC71L | Coiled-Coil Domain Containing 71 Like | 26685 |
| GNA13 | G Protein Subunit Alpha 13 | 4381 |
| STAT4 | Signal Transducer And Activator Of Transcription 4 | 11365 |
| STAT5A | Signal Transducer And Activator Of Transcription 5A | 11366 |
| STX16 | Syntaxin 16 | 11431 |
| STX6 | Syntaxin 6 | 11441 |
| ZBTB34 | Zinc Finger And BTB Domain Containing 34 | 31446 |
| TET1 | Tet Methylcytosine Dioxygenase 1 | 29484 |
| TET3 | Tet Methylcytosine Dioxygenase 3 | 28313 |
| TFDP2 | Transcription Factor Dp-2 | 11751 |
| TGFB2 | Transforming Growth Factor Beta 2 | 11768 |
| TIAM1 | TIAM Rac1 Associated GEF 1 | 11805 |
| FAM168B | Family With Sequence Similarity 168 Member B | 27016 |
| LRRC40 | Leucine Rich Repeat Containing 40 | 26004 |
| MACC1 | MET Transcriptional Regulator MACC1 | 30215 |
| USP53 | Ubiquitin Specific Peptidase 53 | 29255 |
| ZNF148 | Zinc Finger Protein 148 | 12933 |
| RNF145 | Ring Finger Protein 145 | 20853 |
| RNF168 | Ring Finger Protein 168 | 26661 |
| RNMT | RNA Guanine-7 Methyltransferase | 10075 |
| OPA3 | Outer Mitochondrial Membrane Lipid Metabolism Regulator OPA3 | 8142 |
| SECISBP2L | SECIS Binding Protein 2 Like | 28997 |
| PPP1R15B | Protein Phosphatase 1 Regulatory Subunit 15B | 14951 |
| SEPT7 | Septin 7 | 1717 |
| SEPT8 | Septin 8 | 16511 |
| SESTD1 | SEC14 And Spectrin Domain Containing 1 | 18379 |
| SFPQ | Splicing Factor Proline And Glutamine Rich | 10774 |
| SH2B3 | SH2B Adaptor Protein 3 | 29605 |
| PHLPP1 | PH Domain And Leucine Rich Repeat Protein Phosphatase 1 | 20610 |
| RASSF8 | Ras Association Domain Family Member 8 | 13232 |
| PRELID2 | PRELI Domain Containing 2 | 28306 |
| RAP2C | RAP2C, Member Of RAS Oncogene Family | 21165 |
| IBA57 | Iron-Sulfur Cluster Assembly Factor IBA57 | 27302 |
| IGF2BP2 | Insulin Like Growth Factor 2 MRNA Binding Protein 2 | 28867 |
| IPO5 | Importin 5 | 6402 |
| IREB2 | Iron Responsive Element Binding Protein 2 | 6115 |
| RPL36A | Ribosomal Protein L36a | 10359 |
| SCD5 | Stearoyl-CoA Desaturase 5 | 21088 |
| KIAA1549 | KIAA1549 | 22219 |
| SIK1 | Salt Inducible Kinase 1 | 11142 |
| FOXJ3 | Forkhead Box J3 | 29178 |
| FOXN3 | Forkhead Box N3 | 1928 |
| FUS | FUS RNA Binding Protein | 4010 |
| SLC35D1 | Solute Carrier Family 35 Member D1 | 20800 |
| 6-Mar | / | / |
| H2AFZ(H2AZ1) | H2A.Z Variant Histone 1 | 4741 |
| SIRT1 | Sirtuin 1 | 14929 |
| HNRNPF | Heterogeneous Nuclear Ribonucleoprotein F | 5039 |
| HOXB5 | Homeobox B5 | 5116 |
| HIC2 | HIC ZBTB Transcriptional Repressor 2 | 18595 |
| HMGB1 | High Mobility Group Box 1 | 4983 |
| ITGB8 | Integrin Subunit Beta 8 | 6163 |
| THRB | Thyroid Hormone Receptor Beta | 11799 |
| SLC25A3 | Solute Carrier Family 25 Member 3 | 10989 |
| KCTD20 | Potassium Channel Tetramerization Domain Containing 20 | 21052 |
| NAMPT | Nicotinamide Phosphoribosyltransferase | 30092 |
| RAB3D | RAB3D, Member RAS Oncogene Family | 9779 |
| RAB8B | RAB8B, Member RAS Oncogene Family | 30273 |
| PHLPP2 | PH Domain And Leucine Rich Repeat Protein Phosphatase 2 | 29149 |
| RAB10 | RAB10, Member RAS Oncogene Family | 9759 |
| RAB14 | RAB14, Member RAS Oncogene Family | 16524 |
| PRNP | Prion Protein | 9449 |
| SYNCRIP | Synaptotagmin Binding Cytoplasmic RNA Interacting Protein | 16918 |
| PTEN | Phosphatase And Tensin Homolog | 9588 |
| RAB34 | RAB34, Member RAS Oncogene Family | 16519 |
| PSMD9 | Proteasome 26S Subunit, Non-ATPase 9 | 9567 |
| STARD13 | StAR Related Lipid Transfer Domain Containing 13 | 19164 |
| ACVR1 | Activin A Receptor Type 1 | 171 |
| TMEM9B | TMEM9 Domain Family Member B | 1168 |
| TNRC6A | Trinucleotide Repeat Containing Adaptor 6A | 11969 |
| TGIF2 | TGFB Induced Factor Homeobox 2 | 15764 |
| RANGAP1 | Ran GTPase Activating Protein 1 | 9854 |
| SNRPD1 | Small Nuclear Ribonucleoprotein D1 Polypeptide | 11158 |
| CDS1 | CDP-Diacylglycerol Synthase 1 | 1800 |
| CHUK | Component Of Inhibitor Of Nuclear Factor Kappa B Kinase Complex | 1974 |
| CEP55 | Centrosomal Protein 55 | 1161 |
| TMED7 | Transmembrane P24 Trafficking Protein 7 | 24253 |
| DICER1 | Dicer 1, Ribonuclease III | 17098 |
| RAB12 | RAB12, Member RAS Oncogene Family | 31332 |
| ALCAM | Activated Leukocyte Cell Adhesion Molecule | 400 |
| WASL | WASP Like Actin Nucleation Promoting Factor | 12735 |
| WNT10B | Wnt Family Member 10B | 12775 |
| YWHAB | Tyrosine 3-Monooxygenase/Tryptophan 5-Monooxygenase Activation Protein Beta | 12849 |
| ZFPM2 | Zinc Finger Protein, FOG Family Member 2 | 16700 |
| ZFYVE26 | Zinc Finger FYVE-Type Containing 26 | 20761 |
| ZMAT3 | Zinc Finger Matrin-Type 3 | 29983 |
| GNB5 | G Protein Subunit Beta 5 | 4401 |
| GPATCH8 | G-Patch Domain Containing 8 | 29066 |
| SMAD2 | SMAD Family Member 2 | 6768 |
| RPS6KA5 | Ribosomal Protein S6 Kinase A5 | 10434 |
| IKBKB | Inhibitor Of Nuclear Factor Kappa B Kinase Subunit Beta | 5960 |
| IL6ST | Interleukin 6 Cytokine Family Signal Transducer | 6021 |
| ITGA5 | Integrin Subunit Alpha 5 | 6141 |
| JARID2 | Jumonji And AT-Rich Interaction Domain Containing 2 | 6196 |
| KANSL1 | KAT8 Regulatory NSL Complex Subunit 1 | 24565 |
| LCOR | Ligand Dependent Nuclear Receptor Corepressor | 29503 |
| LDLR | Low Density Lipoprotein Receptor | 6547 |
| DDX6 | DEAD-Box Helicase 6 | 2747 |
| DNMT1 | DNA Methyltransferase 1 | 2976 |
| DYNLL2 | Dynein Light Chain LC8-Type 2 | 24596 |
| EFNB2 | Ephrin B2 | 3227 |
| TRIM59 | Tripartite Motif Containing 59 | 30834 |
| USP4 | Ubiquitin Specific Peptidase 4 | 12627 |
| MPP5(PALS1) | Protein Associated With LIN7 1, MAGUK P55 Family Member | 18669 |
| MTMR9 | Myotubularin Related Protein 9 | 14596 |
| BCL2L11 | BCL2 Like 11 | 994 |
| NPTX1 | Neuronal Pentraxin 1 | 7952 |
| INO80 | INO80 Complex ATPase Subunit | 26956 |
| OTUD4 | OTU Deubiquitinase 4 | 24949 |
| PAPD4(TENT2) | Terminal Nucleotidyltransferase 2 | 26776 |
| PBXIP1 | PBX Homeobox Interacting Protein 1 | 21199 |
| PDIA3 | Protein Disulfide Isomerase Family A Member 3 | 4606 |
| PPP6R1 | Protein Phosphatase 6 Regulatory Subunit 1 | 29195 |
| MAFB | MAF BZIP Transcription Factor B | 6408 |
| MAP3K9 | Mitogen-Activated Protein Kinase Kinase Kinase 9 | 6861 |
| ARL6IP1 | ADP Ribosylation Factor Like GTPase 6 Interacting Protein 1 | 697 |
| ARL8B | ADP Ribosylation Factor Like GTPase 8B | 25564 |
| ARRDC3 | Arrestin Domain Containing 3 | 29263 |
| RUFY2 | RUN And FYVE Domain Containing 2 | 19761 |
| SOS2 | SOS Ras/Rho Guanine Nucleotide Exchange Factor 2 | 11188 |
| S1PR1 | Sphingosine-1-Phosphate Receptor 1 | 3165 |
| FXR1 | FMR1 Autosomal Homolog 1 | 4023 |
| BMP3 | Bone Morphogenetic Protein 3 | 1070 |
| BRPF1 | Bromodomain And PHD Finger Containing 1 | 14255 |
| BTBD3 | BTB Domain Containing 3 | 15854 |
| C18orf25 | Chromosome 18 Open Reading Frame 25 | 28172 |
| CAND1 | Cullin Associated And Neddylation Dissociated 1 | 30688 |
| SNAPIN | SNAP Associated Protein | 17145 |
| CCT6A | Chaperonin Containing TCP1 Subunit 6A | 1620 |
| WNT1 | Wnt Family Member 1 | 12774 |
| MAP3K4 | Mitogen-Activated Protein Kinase Kinase Kinase 4 | 6856 |
| EOGT | EGF Domain Specific O-Linked N-Acetylglucosamine Transferase | 28526 |
| GLRX5 | Glutaredoxin 5 | 20134 |
| NEURL4 | Neuralized E3 Ubiquitin Protein Ligase 4 | 34410 |
| ERRFI1 | ERBB Receptor Feedback Inhibitor 1 | 18185 |
| MDM4 | MDM4 Regulator Of P53 | 6974 |
| FAM104A | Family With Sequence Similarity 104 Member A | 25918 |
| GPRC5A | G Protein-Coupled Receptor Class C Group 5 Member A | 9836 |
| POLDIP3 | DNA Polymerase Delta Interacting Protein 3 | 23782 |
| HCCS | Holocytochrome C Synthase | 4837 |
| HSP90B1 | Heat Shock Protein 90 Beta Family Member 1 | 12028 |
| F3 | Coagulation Factor III, Tissue Factor | 3541 |
| MNT | MAX Network Transcriptional Repressor | 7188 |
| MLEC | Malectin | 28973 |
| HOXC8 | Homeobox C8 | 5129 |
| NRAS | NRAS Proto-Oncogene, GTPase | 7989 |
| EPM2AIP1 | EPM2A Interacting Protein 1 | 19735 |
| ARL5B | ADP Ribosylation Factor Like GTPase 5B | 23052 |
| ZNF675 | Zinc Finger Protein 675 | 30768 |
| ADNP2 | ADNP Homeobox 2 | 23803 |
| PPRC1 | PPARG Related Coactivator 1 | 30025 |
| NRBF2 | Nuclear Receptor Binding Factor 2 | 19692 |
| PTPN4 | Protein Tyrosine Phosphatase Non-Receptor Type 4 | 9656 |
| SP3 | Sp3 Transcription Factor | 11208 |
| FOXC1 | Forkhead Box C1 | 3800 |
| UBQLN1 | Ubiquilin 1 | 12508 |
| PAX6 | Paired Box 6 | 8620 |
| RIF1 | Replication Timing Regulatory Factor 1 | 23207 |
| MIER3 | MIER Family Member 3 | 26678 |
| THAP5 | THAP Domain Containing 5 | 23188 |
| ZNF254 | Zinc Finger Protein 254 | 13047 |
| NFIA | Nuclear Factor I A | 7784 |
| EPB41L3 | Erythrocyte Membrane Protein Band 4.1 Like 3 | 3380 |
| ARID1A | AT-Rich Interaction Domain 1A | 11110 |
| IGF1R | Insulin Like Growth Factor 1 Receptor | 5465 |
| ABI2 | Abl Interactor 2 | 24011 |
| ECT2 | Epithelial Cell Transforming 2 | 3155 |
| GAN | Gigaxonin | 4137 |
| CYB5A | Cytochrome B5 Type A | 2570 |
| FOXO3 | Forkhead Box O3 | 3821 |
| KAT6A | Lysine Acetyltransferase 6A | 13013 |
| MTPN | Myotrophin | 15667 |
| LMO2 | LIM Domain Only 2 | 6642 |
| FBXW7 | F-Box And WD Repeat Domain Containing 7 | 16712 |
| FOXO1 | Forkhead Box O1 | 3819 |
| YPEL1 | Yippee Like 1 | 12845 |
| SMARCD1 | SWI/SNF Related, Matrix Associated, Actin Dependent Regulator Of Chromatin, Subfamily D, Member 1 | 11106 |
| NFIB | Nuclear Factor I B | 7785 |
| NLRP3 | NLR Family Pyrin Domain Containing 3 | 16400 |
| KIAA1549L | KIAA1549 Like | 24836 |
| TWF1 | Twinfilin Actin Binding Protein 1 | 9620 |
| UBR5 | Ubiquitin Protein Ligase E3 Component N-Recognin 5 | 16806 |
| CCKBR | Cholecystokinin B Receptor | 1571 |
| CDC27 | Cell Division Cycle 27 | 1728 |
| CFTR | CF Transmembrane Conductance Regulator | 1884 |
| ZNF365 | Zinc Finger Protein 365 | 18194 |
| RRAS2 | RAS Related 2 | 17271 |
| PARD6B | Par-6 Family Cell Polarity Regulator Beta | 16245 |
| TOX | Thymocyte Selection Associated High Mobility Group Box | 18988 |
| SEPT2 | Septin 2 | 7729 |
| MEF2C | Myocyte Enhancer Factor 2C | 6996 |
| RHOB | Ras Homolog Family Member B | 668 |
| CREBZF | CREB/ATF BZIP Transcription Factor | 24905 |
| PRDM1 | PR/SET Domain 1 | 9346 |
| POLR3G | RNA Polymerase III Subunit G | 30075 |
| PTBP2 | Polypyrimidine Tract Binding Protein 2 | 17662 |
| PKNOX1 | PBX/Knotted 1 Homeobox 1 | 9022 |
| PHF19 | PHD Finger Protein 19 | 24566 |
| PLXNA3 | Plexin A3 | 9101 |
| NACC1 | Nucleus Accumbens Associated 1 | 20967 |
| HOXC4 | Homeobox C4 | 5126 |
| NRP2 | Neuropilin 2 | 8005 |
| SREBF2 | Sterol Regulatory Element Binding Transcription Factor 2 | 11290 |
| NMNAT2 | Nicotinamide Nucleotide Adenylyltransferase 2 | 16789 |
| KDELR1 | KDEL Endoplasmic Reticulum Protein Retention Receptor 1 | 6304 |
| VAV2 | Vav Guanine Nucleotide Exchange Factor 2 | 12658 |
| WDR60(DYNC2I1) | Dynein 2 Intermediate Chain 1 | 21862 |
| ZNF721 | Zinc Finger Protein 721 | 29425 |
| POLR3H | RNA Polymerase III Subunit H | 30349 |
| PRKAA2 | Protein Kinase AMP-Activated Catalytic Subunit Alpha 2 | 9377 |
| GNB2 | G Protein Subunit Beta 2 | 4398 |
| SLC9A1 | Solute Carrier Family 9 Member A1 | 11071 |
| SMG5 | SMG5 Nonsense Mediated MRNA Decay Factor | 24644 |
| SOCS1 | Suppressor Of Cytokine Signaling 1 | 19383 |
| CTDSPL | CTD Small Phosphatase Like | 16890 |
| DCLRE1B | DNA Cross-Link Repair 1B | 17641 |
| EARS2 | Glutamyl-TRNA Synthetase 2, Mitochondrial | 29419 |
| EIF5A2 | Eukaryotic Translation Initiation Factor 5A2 | 3301 |
| RCC2 | Regulator Of Chromosome Condensation 2 | 30297 |
| GTF3C4 | General Transcription Factor IIIC Subunit 4 | 4667 |
| ERBB2 | Erb-B2 Receptor Tyrosine Kinase 2 | 3430 |
| CYTH4 | Cytohesin 4 | 9505 |
| RNF219(OBI1) | ORC Ubiquitin Ligase 1 | 20308 |
| AFF4 | AF4/FMR2 Family Member 4 | 17869 |
| DYRK1A | Dual Specificity Tyrosine Phosphorylation Regulated Kinase 1A | 3091 |
| DYNC2H1 | Dynein Cytoplasmic 2 Heavy Chain 1 | 2962 |
| ARIH1 | Ariadne RBR E3 Ubiquitin Protein Ligase 1 | 689 |
| MAP2K6 | Mitogen-Activated Protein Kinase Kinase 6 | 6846 |
| MAPK9 | Mitogen-Activated Protein Kinase 9 | 6886 |
| BCOR | BCL6 Corepressor | 20893 |
| BEX4 | Brain Expressed X-Linked 4 | 25475 |
| BHLHB9 | Basic Helix-Loop-Helix Family Member B9 | 29353 |
| OCRL | OCRL Inositol Polyphosphate-5-Phosphatase | 8108 |
| ATP5G3 | ATP Synthase Membrane Subunit C Locus 3 | 843 |
| PDCD10 | Programmed Cell Death 10 | 8761 |
| PAK2 | P21 (RAC1) Activated Kinase 2 | 8591 |
| PTP4A2 | Protein Tyrosine Phosphatase 4A2 | 9635 |
| SERPINE1 | Serpin Family E Member 1 | 8583 |
| SLC16A1 | Solute Carrier Family 16 Member 1 | 10922 |
| SPPL2A | Signal Peptide Peptidase Like 2A | 30227 |
| SSX2 | SSX Family Member 2 | 11336 |
| SSX2B | SSX Family Member 2B | 22263 |
| HNRNPUL2 | Heterogeneous Nuclear Ribonucleoprotein U Like 2 | 25451 |
| RAB31 | RAB31, Member RAS Oncogene Family | 9771 |
| TM9SF3 | Transmembrane 9 Superfamily Member 3 | 21529 |
| MAP3K5 | Mitogen-Activated Protein Kinase Kinase Kinase 5 | 6857 |
| ZNF608 | Zinc Finger Protein 608 | 29238 |
| PRRC2B | Proline Rich Coiled-Coil 2B | 28121 |
| PRRC2C | Proline Rich Coiled-Coil 2C | 24903 |
| PRR14L | Proline Rich 14 Like | 28738 |
| SCML2 | Scm Polycomb Group Protein Like 2 | 10581 |
| SHROOM1 | Shroom Family Member 1 | 24084 |
| ZBTB43 | Zinc Finger And BTB Domain Containing 43 | 17908 |
| ASCC3 | Activating Signal Cointegrator 1 Complex Subunit 3 | 18697 |
| EFCAB11 | EF-Hand Calcium Binding Domain 11 | 20357 |
| EIF4E | Eukaryotic Translation Initiation Factor 4E | 3287 |
